# De Quervain’s Tendinitis Following Trapeziectomy for the Management of Trapeziometacarpal Arthritis: A Case Report

**DOI:** 10.7759/cureus.70149

**Published:** 2024-09-25

**Authors:** Ahad A Bugis, Moataz Daadour, Rafea I Hakki, Hayat A Khan, Abdullah S Alzahrani

**Affiliations:** 1 Orthopaedic Surgery, Dr. Sulaiman Al Habib Medical Group, Riyadh, SAU; 2 College of Medicine, Prince Sattam bin Abdulaziz University, Riyadh, SAU; 3 College of Medicine, Alfaisal University, Riyadh, SAU

**Keywords:** trapezectomy, tightrope fixation, thumb cmc arthroplasty, thumb cmc, de quervain's tenosynovitis

## Abstract

De Quervain’s tendonitis develops when multiple factors coincide, causing the entrapment of two valuable tendons responsible for the normal range of motion of the thumb. Consequently, symptoms such as pain in the radial side of the wrist and difficulty in grasping and pinching manifest. This case report aims to discuss the presentation of De Quervain’s tendinitis following trapeziectomy with suspensionplasty using the Mini TightRope implant (Arthrex, Inc., Naples, USA) for the management of trapeziometacarpal arthritis.

## Introduction

De Quervain's tendonitis is a condition of abductor pollicis longus (APL) and extensor pollicis brevis (EPB) entrapment due to thickening and myxoid degeneration of their respective tendon sheaths [1]. The aforementioned tendons are responsible for movements within the range of thumb motion [2]. De Quervain’s tendonitis may indicate an acute inflammatory process. However, myxoid degeneration, in conjunction with fibrous tissue deposits, is believed to be the cause [1]. This condition ordinarily affects females, whether during late pregnancy or the postpartum period. In this case, the patient developed De Quervain’s tendonitis after trapeziectomy for the management of trapeziometacarpal arthritis. Osteoarthritis of the trapeziometacarpal joint (carpometacarpal joint of the first metacarpal, CMC-1) is a common, disabling, degenerative condition [3] that presents as pain, stiffness, weakness, and restricted arc-like motion of the thumb and fingers [4]. It is the most predominant operating condition of the upper extremities in patients with arthritis. Females and postmenopausal women transcend any other population under this condition [2]. The susceptibility to this condition is believed to be attributable to a combination of ligamentous laxity, increased articular surface concavity, and disturbed hormonal homeostasis [5].

## Case presentation

A 56-year-old female presented to our outpatient orthopaedic clinic with chronic progressive left thumb pain, accompanied by pinching and grasping difficulties. The patient denied having any neurological symptoms. Local physical examination and assessment revealed an obvious fixed hyperextension deformity of the thumb. Radiographic evaluation with hand and wrist radiography revealed end-stage (IV) trapeziometacarpal arthritis with pan-trapezial involvement based on the Eaton and Littler classification [6].

**Figure 1:**
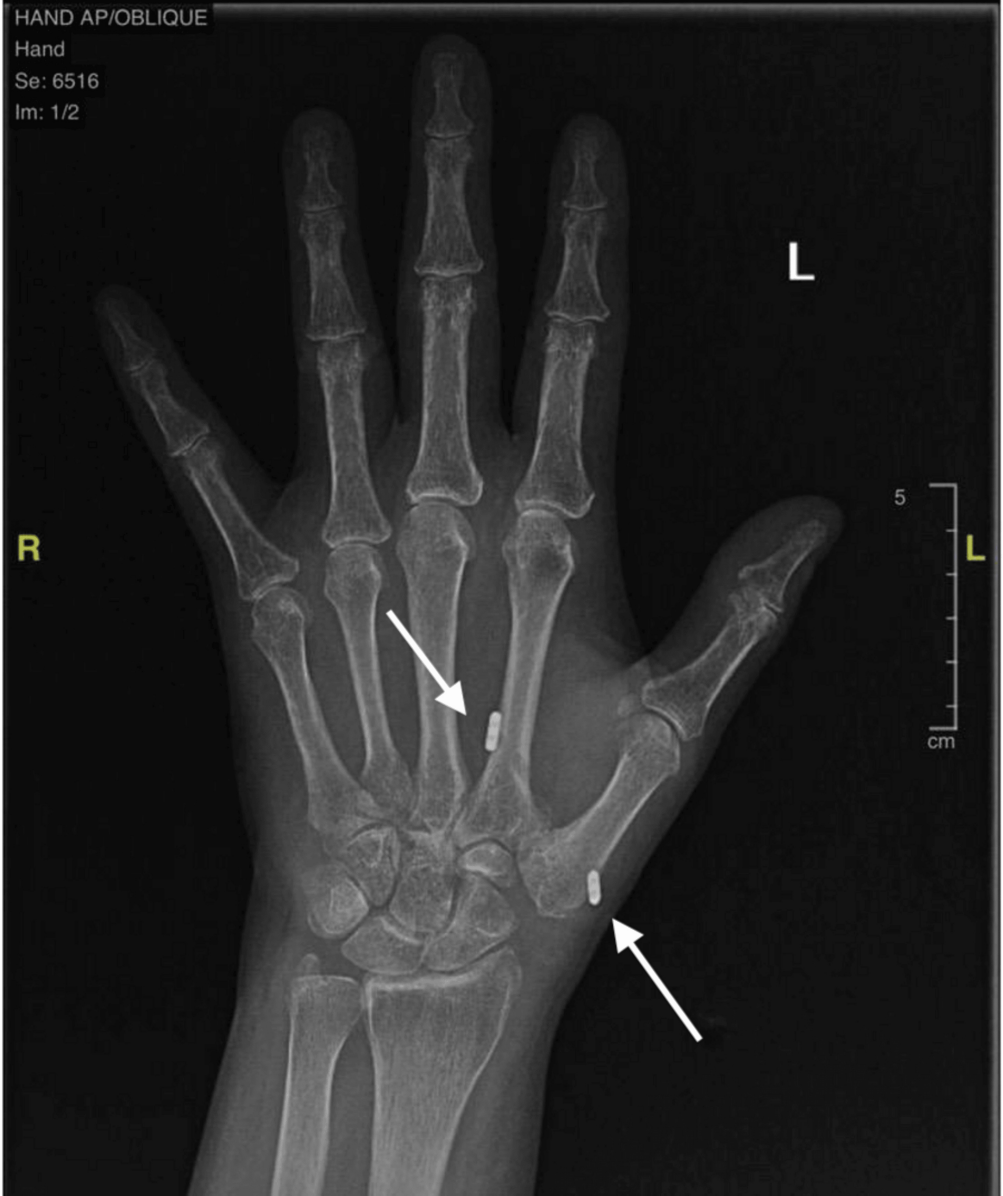
Anteroposterior (AP) X-ray of the hand following trapeziectomy with Mini TightRope fixation, with arrows indicating the Mini TightRope fixation points.

The patient was initially managed conservatively with nonsteroidal anti-inflammatory drugs (NSAIDs) and physiotherapy for one year and eight months, respectively. Consequently, she elected to undergo surgical intervention as the pain was resistant to the conservative approach and continued to affect her activities of daily living (ADLs). Intraoperatively, using a dorsal approach to the left thumb, severe first carpometacarpal arthritis with osteophytes was confirmed and trapeziectomy with Mini TightRope fixation (Arthrex, Inc., Naples, USA) was performed (Figure 1). Upon initial postoperative office follow-up, the patient improved with no complaints and her physiotherapy sessions persisted.

During later follow-ups, approximately six months later, the patient reported radial-sided wrist pain exacerbated by gripping and object raising with the wrist in a neutral position. Physical examination revealed a normal joint range of motion and unremarkable neurological findings. However, the patient had a positive, provocative Finkelstein’s sign. A literature review evaluated the relationship between CMC arthritis, De Quervain’s tendinitis, and trapeziometacarpal arthroplasty. Ledoux found that De Quervain’s tendinitis is an integral part of osteoarthritis of the thumb, both preoperatively and postoperatively [7].

Wrist musculoskeletal ultrasound was instrumental in confirming the thickening of tenosynovitis in the first dorsal extensor compartment, specifically involving the EPB and APL tendons. Conservative management with painkillers and physiotherapy was initiated to confirm the diagnosis of De Quervain’s tendinitis. Upon subsequent follow-up, the symptoms of De Quervain’s tendinitis improved with no complaints.

## Discussion

There are few published articles discussing De Quervain’s tenosynovitis following trapeziectomy. Ledoux found that De Quervain's tendinitis is an integral part of osteoarthritis of the thumb, both preoperatively and postoperatively [7]. Amadio et al. conducted a retrospective study that examined the outcomes after trapezium resection arthroplasty and found that in the non-implant group, three patients developed De Quervain's tenosynovitis after surgery. Further examination revealed that the shortened and ruptured abductor tendon slips were too bulky to pass smoothly through the first dorsal compartment, resulting in inflammation [8]. However, in our case, a 56-year-old Middle Eastern underwent a trapeziectomy with suspensionplasty using the Mini TightRope implant, which helps stabilize the thumb and index finger by keeping their metacarpals aligned [9]. Six months later, throughout her follow-ups in our outpatient clinic alongside physiotherapy sessions, our patient complained of progressive wrist pain and weakness in her daily grip. She began experiencing radial-sided wrist pain exacerbated by gripping and object raising with the wrist in a neutral position. Upon examination, a positive provocative Finkelstein test result was observed, and the diagnosis of De Quervain's tendinitis was confirmed by ultrasound. The patient was prescribed NSAIDs and was re-educated about physiotherapy session compliance over a period of two months, with significant improvement in regaining full strength in the palmar grip. This supports the findings of Goubau et al., which suggest that the cause is not biomechanical [10]. Postoperative care included a forearm cast for three weeks, followed by autogenous rehabilitation.

To date, we posit that the pathophysiology of De Quervain's tendinitis following trapeziectomy involves a combination of factors. Adjacent extensor tendons are conceivably affected by global inflammatory arthritic phenomena. Gómez-Garrid et al. reported a high rate of complications of De Quervain's tenosynovitis (21%) after total joint arthroplasty for managing trapeziometacarpal osteoarthritis and a high chance of recovery with conservative management, usually occurring around the third-month post-surgery [11]. After surgical removal of the trapezium bone, the remaining wrist bones may experience increased stress as they compensate for this loss. This can lead to increased tension on the tendons at the base of the thumb. Additionally, the surgical procedure itself can result in the formation of scar tissue, which may irritate and inflame the tendons. Finally, post-surgical inflammation, a common occurrence after any surgery, can further contribute to tendon irritation and the development of De Quervain's tendinitis, as observed in our patient. The complex interplay between these two hand conditions warrants further investigation to elucidate potential shared risk factors or biomechanical influences. Understanding this relationship may lead to improved diagnostic approaches and more comprehensive treatment strategies for patients with concurrent symptoms. Future research should focus on longitudinal studies to determine if one condition predisposes individuals to another, or if it commonly co-occurs due to underlying anatomical or functional factors.

Goubau et al. [10] and Gómez-Garrido et al. [11] have opted to manage the patients who developed De Quervain’s tendinitis post-operatively with a conservative approach as we did with our patient consisting of either physiotherapy, splints, painkillers or a combination of the listed managements. This supports that De Quervain's tendinitis following trapeziectomy can be managed conservatively (Table 1).

**Table 1:** Comparison of reported studies on De Quervain’s tenosynovitis by age, procedure performed, outcome, management, and incidence rate.

Author	Goubau et al. [10]	Amadio et al. [8]	Gómez-Garrido et al. [11]
Number of Patients	83 patients; 69 females, 12 males, 8 bilateral	50 patients; all females	137 patients; 35 males, 102 females
Age	From 33 to 88 years	From 35 to 75 years old	From 53 to 84 years
Procedure	Ball-and-socket arthroplasty	Trapeziectomy with silicone replacement (25 females); Trapeziectomy without silicone replacement (25 females)	Modified Ceruso’s suspensionplasty following trapeziectomy
Outcome	Fourteen cases (17%) developed De Quervain’s tendinopathy within the first year post-surgery.	In the non-implant group, three patients developed De Quervain’s tenosynovitis postoperatively.	The most frequent postoperative complication was De Quervain’s tenosynovitis (21%).
Management	Conservative management (corticosteroid infiltration and splinting) for 11 cases, while three required surgical intervention.	All three patients were relieved of their symptoms after release of the dorsal retinacular ligament.	All cases were resolved with splinting and physical therapy.

## Conclusions

De Quervain’s tendonitis, a debilitating condition affecting day-to-day activities, may develop following trapeziectomy for trapeziometacarpal arthritis. This may be due to multiple factors including the strenuous effect on adjacent tendons caused by the inflammatory process of arthritis, formation of scar tissue, and post-surgical inflammation. Literature found that joint arthroplasty for trapeziometacarpal arthritis is usually accompanied by a higher rate of complication while on the other hand a higher chance of recovery with a conservative approach. Suitable recognition and management play a significant role in recovery. Further studies focusing on the surgical population are vital to survey the incidence of these complications and to explore preventative measures.
